# Long-term persistence of serum IgM antibodies against chikungunya virus in patients with chronic arthralgia

**DOI:** 10.1186/s12985-025-02721-x

**Published:** 2025-04-24

**Authors:** Leile Camila Jacob-Nascimento, Rosângela O. Anjos, Moyra M. Portilho, Viviane M. Cavalcanti, Adriane S. Paz, Lorena G. Santos, Moisés S. Sousa, Julia G. Costa, Mariane R. Silva, Patrícia S. S. Moreira, Uriel Kitron, Scott C. Weaver, Mittermayer B. Santiago, Mitermayer G. Reis, Guilherme S. Ribeiro

**Affiliations:** 1 Fundação Oswaldo Cruz, Salvador, Brazil; 2 Escola Bahiana de Medicina e Saúde Pública, Salvador, Brazil; 3 Emory University, Atlanta, USA; 4 World Reference Center for Emerging Viruses and Arboviruses, University of Texas Medical Branch, Galveston, USA; 5 Faculdade de Medicina, Universidade Federal da Bahia, Salvador, Brazil; 6 Yale University, New Haven, USA

**Keywords:** Chikungunya, Serology, IgM antibody, Diagnosis, Surveillance

## Abstract

**Background:**

Anti-Chikungunya virus (CHIKV) IgM antibodies may persist for months after infection in some individuals, but the evidence is limited, and their exact duration remains unknown.

**Objective:**

This study aimed to determine the duration for which anti-CHIKV IgM antibodies remain detectable following acute infection.

**Methods:**

A commercial ELISA was used to assess the frequency of anti-CHIKV IgM antibody detection over time in 145 longitudinal serum samples obtained from 45 laboratory-confirmed chikungunya patients in Brazil (two to six samples per patient).

**Results:**

Among samples obtained within seven days post-symptom onset (DPSO), 13% (6/45) were IgM-positive. Between 10 and 120 DPSO, 100% (62/62) of samples were positive. Positivity rates for samples collected between 121 – 720, 721–900, 901–1,080, 1,081–1,260, and > 1,260 DPSO were 62% (5/8), 35% (6/17), 12% (1/8), 33% (1/3) and 50% (1/2), respectively. Notably, among 21 patients who developed chronic arthralgia and had at least one sample collected > 720 DPSO, 7 (33%) still had detectable anti-CHIKV IgM. This suggests that approximately one-third of chikungunya patients with chronic arthralgia may maintain anti-CHIKV IgM for over two years following acute disease.

**Conclusions:**

Our findings indicate that anti-CHIKV IgM antibodies can persist substantially longer than typically observed for acute RNA virus infections. This has significant implications for chikungunya diagnosis and surveillance. Further research is needed to determine whether long-term IgM persistence also occurs in patients without chronic chikungunya symptoms.

## Background

Chikungunya virus (CHIKV) is a vector-borne, single-stranded RNA virus that belongs to the *Togaviridae* family and the *Alphavirus* genus. During the twenty-first century, CHIKV emerged as a significant global health concern, spreading to new tropical and subtropical regions worldwide and causing large epidemics [1]. Most CHIKV infections are symptomatic, manifesting as fever, headache, rash, myalgia, swollen joints, and especially arthralgia, which tends to be severe and can evolve chronically, lasting months to years after infection [2]. Its pathophysiology involves B cell activation and the production of specific neutralizing antibodies [3, 4]. Although the exact duration of detectable antibodies is not fully understood, IgM antibodies are typically expected to remain in the bloodstream for about three months after most acute RNA viral infections, whereas IgG antibodies can persist for several years or even for life [5].

The laboratory diagnosis of chikungunya is primarily performed through the detection of CHIKV RNA by reverse transcription polymerase chain reaction (RT-PCR) or through serological techniques to identify antibodies against CHIKV, such as immunosorbent assay (ELISA) and rapid immunochromatographic tests. An acute CHIKV infection is typically confirmed by a CHIKV-positive RT-PCR in an acute-phase serum sample (obtained 0–7 days post-symptom onset [DPSO]) or by the detection of anti-CHIKV IgM seroconversion between acute- and convalescent-phase (> 7 DPSO) serum samples. In contrast, while detecting anti-CHIKV IgM antibody in an acute-phase sample is commonly used to support an acute infection, it may occasionally represent a prior, recent (but not acute) infection [6–8]. Lastly, concurrently detecting anti-CHIKV IgG and IgM might suggest a recent infection, while the sole presence of IgG may indicate a non-recent past infection [8].

However, a few studies have suggested that IgM antibodies against CHIKV can be detectable after three [9] and even up to 13 years after CHIKV infection [10]. Given that IgM-based serological methods are widely used for chikungunya diagnosis, the findings that anti-CHIKV IgM can persist for long periods raise concerns regarding the correct interpretation of a positive IgM-based test, as it may represent a previous infection rather than an acute or recent infection, especially in settings where large CHIKV epidemics have occurred or endemic CHIKV transmission has been established. Therefore, this study aimed to assess the frequency of IgM and IgG antibody detection over time in serum samples from a cohort of laboratory-confirmed chikungunya patients from Salvador, Brazil.

## Methods

### Participants selection

The patients included in this investigation had the laboratory diagnosis of acute CHIKV infection made during their participation in a surveillance study to detect arbovirus infection. The patients enrolled sought healthcare due to an acute febrile or exanthematous illness in an emergency facility in Salvador, Brazil. Although the surveillance study has been ongoing since January 2009, all the chikungunya patients included in this investigation were diagnosed between June 2019 and March 2020, when large epidemics spread throughout Salvador [11]. The inclusion criteria for the surveillance study during this period were age ≥ 6 months at surveillance enrollment and having an axillary temperature ≥ 37.8 °C or reported fever or skin rash lasting ≤ 7 days.

During surveillance enrollment, an interview was conducted to obtain clinical and epidemiological data, and an acute-phase blood sample was collected by venipuncture. Participants were invited to return 10–45 days later to report on the evolution of symptoms and to collect a convalescent-phase blood sample. If the participant could not return to the health unit, a research team attempted to collect the convalescent-phase clinical data and blood sample during a visit to the participant's home. Interview data were recorded in an electronic form and were automatically transferred into a database using the REDCap (Research Electronic Data Capture) software [12]. After collection, blood samples were refrigerated (2–8 °C) at the health unit until cold transportation to our laboratory at Fundação Oswaldo Cruz on the same day. There, they were centrifuged, and the sera were aliquoted and stored at −20 °C and −80 °C until being tested by serological and molecular methods. Additional details on the long-term surveillance study had been previously reported [13–15].

### Laboratory diagnosis

Participants enrolled in the surveillance study had the diagnosis of an acute CHIKV infection confirmed based on (1) a positive result for CHIKV in the Trioplex qRT-PCR, which can detect RNA from CHIKV, ZIKV, and DENV according to the protocols from the Centers for Disease Control and Prevention [16]; or (2) detection of CHIKV IgM seroconversion between acute- and convalescent-phase serum samples using the CHIKjj Detect™ IgM ELISA kit (InBios International, Seattle, United States). In addition, all participants in the surveillance study were also tested by ELISA for the detection of anti-DENV IgM antibodies (Panbio Diagnostics, Brisbane, Australia) and DENV NS1 antigen (Panbio Diagnostics, Brisbane, Australia). All assays were performed following the manufacturer's instructions.

### Chikungunya patients'follow-up

Participants with a laboratory-confirmed acute CHIKV infection were contacted by telephone and invited for an outpatient follow-up at the rheumatology service of the Universidade Federal da Bahia Hospital complex. Those interested in the follow-up had an appointment with a rheumatologist scheduled and, during the consultation, were examined, oriented, and treated based on Brazilian guidelines for chikungunya management [7, 17]. Patients had regular return appointments scheduled as indicated by the rheumatologist, according to the patient’s needs. Data on disease signs and symptoms, physical exams, and medical care were obtained during these visits using the Research Electronic Data Capture (REDCap) software. Blood samples were also collected, refrigerated, and transported on the same day to the Oswaldo Cruz Foundation laboratory, where they were processed, aliquoted, and stored at −80 °C until testing.

### Detection of IgM and IgG antibodies against CHIKV

The final group of participants included in this study comprised laboratory-confirmed chikungunya cases identified during the surveillance study between June 2019 and March 2020 who attended the rheumatology outpatient clinic and had at least one blood sample collected during the rheumatological follow-up. Although collecting a convalescent-phase sample during the surveillance study was not mandatory, only one patient failed to provide it. Additionally, two patients had both the convalescent phase and first outpatient clinic samples collected within 10–30 days after infection, but only the most recent sample was considered, as they were from the same time window.

The same CHIKV IgM ELISA used in the surveillance study was used to determine the presence of anti-CHIKV antibodies in follow-up samples obtained during outpatient appointments. IgG antibodies against CHIKV were also assessed in all participants'serum samples using the IgG ELISA from Euroimmun, according to the manufacturer’s instruction (Euroimmun, Luebeck, Germany). Finally, all the samples studied – those collected at the acute and convalescent phases as part of the surveillance study and those collected during rheumatological follow-up – underwent the same Trioplex qRT-PCR protocol used in the surveillance study. This aimed to determine how long they remained CHIKV RNA-positive and whether this positivity was associated with IgM positivity.

### Data analysis

All clinical data and patient laboratory results were stored in the REDCap digital database [12] before being exported and analyzed using STATA version 13, Microsoft Excel and Prism version 7 software. Clinical, demographic, and laboratory characteristics were described using absolute and relative frequencies for categorical variables and medians and minimum–maximum or interquartile (IQR) ranges for continuous variables. The positivity rates for the presence of anti-CHIKV IgM and IgG, and CHIKV RNA detected by RT-qPCR, were calculated as relative frequencies for the following periods: 0–7, 10–30, 31–60, 61–90, 91–120, 121–720, 721–900, 901–1,080, 1,081–1,260, and > 1,260 DPSO. These periods were arbitrarily defined based on the availability of serum samples within each period. The positivity rate for the detection of anti-CHIKV IgM was also calculated for the patients with at least one sample collected > 720 DPSO. Individuals with missing information for any variable were excluded from the analysis. Lastly, we used frequencies or medians and interquartile ranges to compare demographic, clinical, and laboratory characteristics between the following groups of patients: those with IgM maintenance for > 720 DPSO, those without IgM maintenance for > 720 DPSO, and those not evaluated after 720 DPSO due to lack of sample collection. P-values were calculated to assess statistical differences for these comparisons, using the Kruskall-Wallis test for quantitative variables and Fisher’s exact test for categorical variables. However, given the small sample size per group and the exploratory nature of this comparison, P-values should be interpreted with caution due to limited statistical power.

### Ethical approval

This study was approved by the Institutional Review Board of the Instituto Gonçalo Moniz, Fundação Oswaldo Cruz (CAAE: 55,904,616.4.0000.0040). Written informed consent was used during both enrollment in the surveillance study and the first consultation at the rheumatological clinic to explain the study’s aims, procedures, risks, and benefits. The forms were signed by participants ≥ 18 years old and parents of participants < 18. Minors (aged 5–17) also signed an informed assent form.

## Results

Between June 2019 and March 2020, 1,039 patients with an acute febrile or exanthematous illness were enrolled in the surveillance study, of whom 395 were diagnosed with acute chikungunya; 382 (96.7%) by RT-qPCR with or without detection of IgM seroconversion, and 13 (3.3%) by IgM seroconversion alone. Although telephone contact attempts were made for 300 of the 395 patients, only 82 responded, and 47 accepted the invitation for a follow-up visit at the rheumatological clinic and attended at least once. Of those 47 visiting the clinic, 45 had at least one additional blood sample collected, comprising the group of patients of this investigation.

These 45 patients attended the outpatient rheumatological clinic between October 2019 and April 2023. The median, minimum, and maximum intervals from symptom onset and the last clinic visit were 170, 29, and 1,316 days, respectively. Their diagnosis of CHIKV infection was based on both positive RT-qPCR and IgM seroconversion in 36 (80.0%) patients, solely on a positive RT-qPCR in 7 (15.6%) patients, 6 of whom also had a positive IgM on the first sample, and one who did not have a convalescent-phase serum sample for seroconversion evaluation, and solely on IgM seroconversion in 2 (4.4%) patients. Most (33; 73.3%) patients were women and their median age was 44 (IQR: 35–56; min–max range: 21–79) years (Table 1). All patients reported acute arthralgia during enrollment in the surveillance study. Thirty of them developed chronic arthralgia, defined by joint pain lasting > 90 days [21]. The other 15 patients were followed for less than 90 days, preventing an assessment of whether they developed chronic arthralgia. However, all these 15 patients still experienced joint pain in their last appointment (between 29 and 84 DPSO). Arthralgia had an intermittent nature, with some patients reporting periods without joint pain during a few visits (Table 1).

**Table 1:** Demographic, clinical and laboratory features of laboratory-confirmed chikungunya patients

Characteristics	Days post symptoms onset (DPSO) at the time of the visit									
	0–7 (*N* = 45)	10–30 (*N* = 40)	31–60 (*N* = 13)	61–90 (*N* = 7)	91–120 (*N* = 2)	121–720 (*N* = 8)	721–900 (*N* = 17)	901–1080 (*N* = 8)	1,081–1,260 (*N* = 3)	> 1,260 (*N* = 2)
Demographics and clinical	% (n) or median (interquartile range)									
Sex (female)	33 (73)	30 (75)	11 (84)	4 (57)	1 (50)	7 (87)	12 (70)	6 (75)	2 (66)	1 (50)
Age	44 (35–56)	45 (35–56)	37 (30–49)	56 (47–52)	38 (33–44)	54 (40–68)	44 (33–52)	40 (33–48)	37 (21–55)	44 (33–56)
DPSO	1 (1.5–3)	15 (13–20)	36 (37–44)	74 (67–78)	102 (96 −107)	166 (147–271)	848 (806–860)	1008 (959–1,022)	1,120 (1,120–1172,5)	1,301.5 (1,294–1308,8)
Arthralgia	45 (100)	35 (94) ^*a*^	12 (92)	7 (100)	2 (100)	7 (87)	17 (100)	8 (100)	3 (100)	2 (100)
Myalgia	45 (100)	0 (0) ^*b*^	8 (80) ^*d*^	6 (85)	0 (0)	4 (50)	13 (76)	5 (62)	2 (66)	1 (50)
Joint edema	31 (68)	27 (75) ^*c*^	12 (100) ^*e*^	7 (100)	2 (100)	5 (62)	12 (75)	7 (87)	2 (66)	2 (100)
*Laboratory*										
RT-qPCR CHIKV positive	43 (95)	5 (2)	0 (0)	0 (0)	0 (0)	0 (0)	0 (0)	0 (0)	0 (0)	0 (0)
Cycle Threshold	22.82 (20.0–28.4)	36.75 (35.6–37.9)	–	–	–	–	–	–	–	–
ELISA IgM positive ^*f,h*^	6 (13)	40 (100)	13 (100)	7 (100)	2 (100)	5 (62)	6 (35)	1 (12)	1 (33)	1 (50)
ELISA IgG positive ^*g*^^*,h*^	1 (2)	40 (100)	13 (100)	7 (100)	2 (100)	8 (100)	17 (100)	8 (100)	3 (100)	2 (100)

^*a*^ Data available for 37 patients

^*b*^ Data available for 2 patients

^*c*^ Data available for 36 patients

^*d*^ Data available for 10 patients

^*e*^ Data available for 12 patients

^*f*^ Results based on the sample-to-calibrator optical density ratio for the IgM ELISA are interpreted as follows: Negative < 0.9; Positive > 1.1; Indeterminate ≥ 0.9 and ≤ 1.1

^*g*^ Results based on sample-to-calibrator optical density ratio for the IgG ELISA are interpreted as follows: Negative < 0.8; Positive ≥ 1.1; Indeterminate ≥ 0.8 and < 1.1

^*h*^ The Sample/Calibrator ratio is calculated by dividing the optical density obtained with the test sample by the calibrator value, which is unique for the IgG ELISA and an average of a duplicate calibrator for the IgM ELISA

Anti-CHIKV antibodies were investigated in the acute-phase serum samples from all 45 patients, in the convalescent-phase samples collected during the surveillance study from 42 (93.3%) patients, and in at least one additional sample collected during the rheumatological follow-up. Overall, 145 samples were tested, and the median, minimum, and maximum number of serum samples tested per patient were 3, 2, and 6, respectively.

**Fig. 1:**
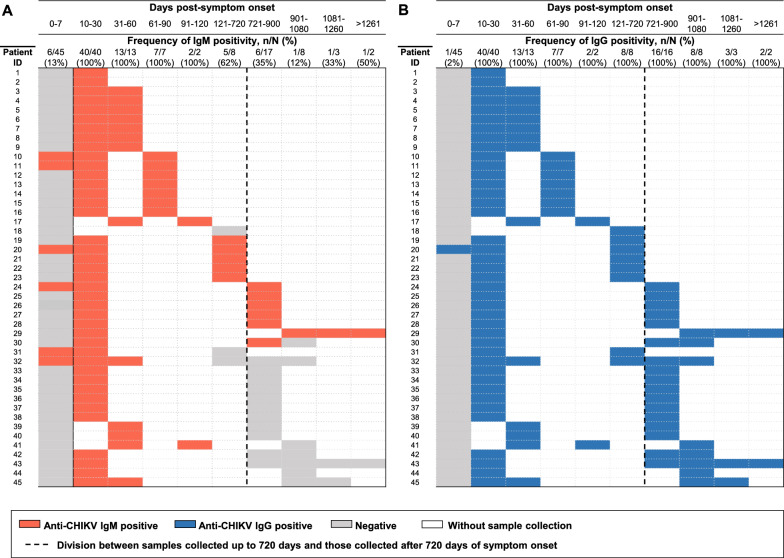
Frequency of anti-CHIKV **A** IgM and **B** IgG detection in chikungunya patients by days post-symptom onset

Among the 45 acute-phase samples collected within 7 DPSO, only 6 (13%) tested positive for anti-CHIKV IgM antibodies (Table 1; Fig. 1A). In contrast, all 62 samples collected from these 45 patients between 10 and 120 DPSO were IgM-positive. For the eight patients with serum samples obtained between four months and two years after symptoms onset (121–720 DPSO), 5 (62%) remained IgM-positive. For the 21 patients with at least one serum sample collected after two years of symptoms onset (> 720 DPSO), 7 (33%) had detectable anti-CHIKV IgM (Fig. 1A). Notably, one patient's serum sample obtained 1,287 DPSO was still IgM positive (Fig. 1A, Patient ID 29). Despite being RT-qPCR positive and showing IgG seroconversion, one patient did not have an IgM seroconversion detected, but their unique follow-up sample was obtained 133 DPSO (Fig. 1, Patient ID 18).

IgG seroconversion occurred for 44 (98%) of 45 patients (Table 1; Fig. 1B). The exception was an RT-qPCR-positive patient whose first sample, collected 0–7 DPSO, was already positive for both IgM and IgG (Fig. 1B, Patient ID 20). Anti-CHIKV IgG antibodies were detected in all follow-up samples from the 45 patients. In addition to the 43 acute-phase samples that were RT-qPCR positive for CHIKV, only two other samples, collected at 13 and 16 DPSO, were positive for CHIVK by RT-qPCR, with Ct values of 35.6 and 37.6, respectively. None of the 60 samples collected after 30 DPSO were RT-qPCR positive for CHIKV.

**Fig. 2:**
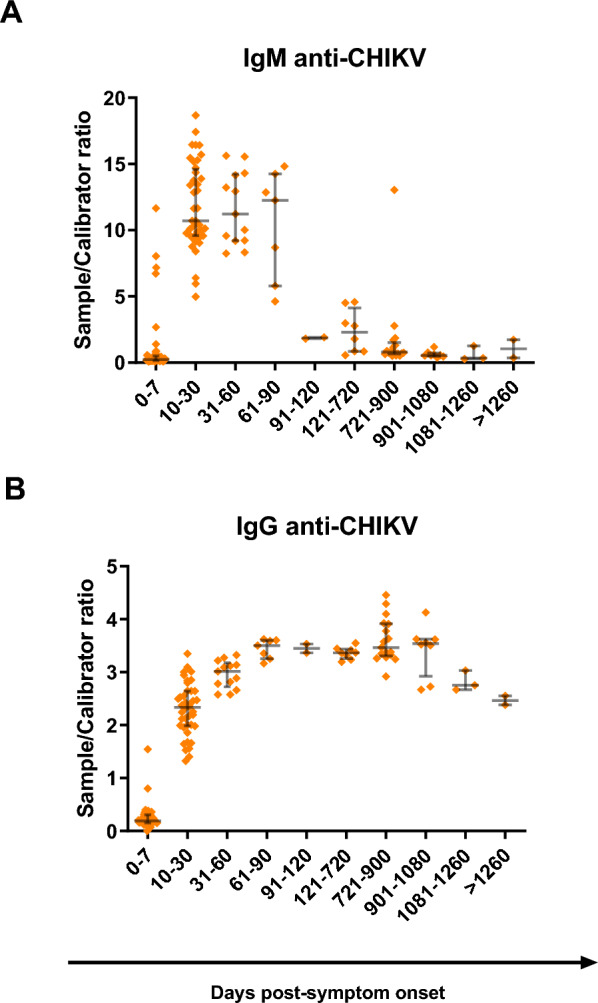
Evolution of anti-CHIKV antibody responses in relation to symptom onset in chikungunya patients. **A** Kinetics of anti-CHIKV IgM by days post-symptom onset. **B** Kinetics of anti-CHIKV IgG by days post-symptom onset

We used the ratios between the ODs of the tested sample and the assay calibrator to estimate the amount of anti-CHIKV antibodies in each sample (Fig. 2). We observed a sharp increase in the anti-CHIKV IgM levels when comparing the samples obtained 0–7 DPSO to those obtained 10–30 DPSO (Fig. 2A). This was followed by a gradual increase, with IgM levels peaking in samples collected 61–90 DPSO. Afterward, there was a steep decline in IgM levels in later samples. This pattern mirrored the trend in IgM positivity rates over time. Anti-CHIKV IgG levels also increased when comparing the median OD/calibrator ratios between samples obtained 0–7 DPSO and 10–30 DPSO (Fig. 2B). The increase continued through the 31–60 DPSO samples, reaching a peak in the 61–90 DPSO samples. However, unlike the IgM levels, IgG levels remained relatively stable in follow-up samples up to 901–1,080 DPSO. A gradual decline in IgG levels was observed in the subsequent samples, although their levels remained higher than those in the 10–30 DPSO group.

To explore whether demographic and initial clinical and laboratory characteristics were related to the long-term persistence of anti-CHIKV IgM, we compared these characteristics among three groups: 7 patients with IgM persistence for > 720 DPSO, 14 patients without IgM persistence for > 720 DPSO, and 24 patients not evaluated after 720 DPSO due to insufficient sample collection (Table 2). Patients with IgM persistence were older (median 52 years; IQR: 34–56) than those without persistence (median 43; IQR 32–47) or not evaluated after 720 DPSO (median 44; IQR 31–67) (*P* = 0.41). Patients with IgM persistence also had a higher frequency of joint edema (85%) than those without persistence (64%) or not evaluated (66%) (*P* = 0.74). Conversely, the frequency of skin rash was lower in the group with IgM persistence (28%) compared to those without persistence (42%) or not evaluated (43%) (*P* = 0.83). Positivity in the CHIKV RT-qPCR performed in the initial sample was observed for 71% of the patients with IgM persistence, compared to 100% in the other two groups (*P* = 0.02) and the median RT-qPCR CT level for the positive samples was slightly lower in the group with IgM persistence (20.68) compared to the other group of patients (22.49 and 24.97, respectively) (*P* = 0.08). No substantial differences were observed among groups in the frequency of comorbidities or the levels of anti-CHIKV IgM and IgG early in the course of the illness (at 0–7 and 10–30 DPSO).

**Table 2:** Acute-phase clinical and laboratory features of patients followed > 720 days post-symptom onset by IgM status

Characteristics	Anti-CHIKV IgM persistence > 720 days (*n* = 7)	Anti-CHIKV IgM persistence ≤ 720 days (*n* = 14)	Without sample collection > 720 days (*n* = 24)	Total (*N* = 45)	*P *Value^*a*^
	n (%) or median (interquartile range)				
*Demographic*					
Sex (female)	5 (71)	9 (64)	19 (79)	33 (73)	0.59
Age	52 (37–56)	43 (32–47)	44 (31–67)	44 (35–56)	0.41
*Acute chikungunya clinical manifestations*					
Fever	7 (100)	13 (93)	24 (100)	44 (97)	0.46
Arthralgia	7 (100)	14 (100)	24 (100)	45 (100)	-
Myalgia	7 (100)	14 (100)	24 (100)	45 (100)	-
Joint edema ^*b*^	6 (85)	9 (64)	16 (66)	31 (68)	0.74
Rash	2 (28)	6 (42)	10 (43) ^*c*^	18 (41) ^*d*^	0.83
Pruritus	2 (28)	5 (33)	10 (41)	17 (37)	0.91
Abdominal pain	5 (71)	9 (64)	7 (29)	21 (46)	0.03
Vomit	1 (14)	2 (14)	8 (33)	11 (24)	0.45
*Prior medical conditions*					
Pre-existing joint pain	3 (42)	6 (42)	7 (35)	16 (35)	0.62
Hypertension	2 (28)	4 (28)	8 (33)	14 (31)	1.00
Diabetes	1 (14)	2 (14)	1 (4)	4 (8)	0.46
*Laboratory results, 0–7 DPSO*					
Anti-CHIKV IgM positive ^*e,,f*^	1 (14)	1 (7)	4 (16)	6 (13)	0.84
Sample OD/calibrator ratio	0.27 (0.16–0.44)	0.20 (0.15–0.43)	0.21 (0.15–0.55)	0.23 (0.16–0.48)	0.90
Anti-CHIKV IgG positive ^*e,g*^	0 (0)	0 (0)	1 (4)	1 (2)	1.00
Sample OD/calibrator ratio	0.18 (0.18–0.30)	0.20 (0.16–0.31)	0.19 (0.14–0.28)	0.19 (0.15–0.30)	0.85
RT-qPCR CHIKV positive	5 (71)	14 (100)	24 (100)	43 (95)	0.02
Cycle threshold	20.68 (20.4–22.82)	22.49 (20.05–27.66)	24.97 (20.43–33.23)	22.96 (20.06–30.51)	0.08
*Laboratory results, 10–30 DPSO*	*n* = 7	*n* = 11	*n* = 22	*n* = 40	
Anti-CHIKV IgM positive ^*c,d*^	7 (100)	11 (100)	22 (100)	40 (100)	-
Sample OD/calibrator ratio	11.63 (10.12–16.43)	13.40 (9.71–13.88)	10.19 (9.44–15.18)	10.71 (9.61–14.52)	0.68
Anti-CHIKV IgG positive ^*e,g*^	7 (100)	11 (100)	22 (100)	40 (100)	-
Sample OD/calibrator ratio	2.24 (2.11–3.09)	2.34 (1.95–2.49)	2.35 (1.98–2.64)	2.33 (1.98–2.64)	0.93
RT-qPCR CHIKV positive	0 (0)	2 (18)	0 (0)	2 (5)	0.69
Cycle threshold	–	36.75 (35.6–37.9)	–	36.75 (35.6–37.9)	–

^*a*^* P-values were calculated using the Kruskall-Wallis test for quantitative variables and Fisher’s exact test for categorical variables*

^*b*^* This variable refers to edema in at least one of the following joints: fingers, toes, elbows, wrists, knees and ankles*

^*c*^* Data available for 23 patients*

^*d*^* Data available for 44 patients*

^*e*^* Sample/calibrator ratio is calculated from the ratio of the optical density obtained with the test sample divided by the calculated calibrator value*

^*f*^* Results based on sample/calibrator ratio for IgM ELISA are interpreted as follows:* < *0.9* = *Negative;* > *1.1* = *Positive;* ≥ *0.9 and* ≤ *1.1* = *Indeterminate*

^*g*^* Results based on sample/calibrator ratio for IgG ELISA are interpreted as follows:* < *0.8* = *Negative;* ≥ *1.1* = *Positive;* ≥ *0.8 and* < *1.1* = *Indeterminate*

## Discussion

Our findings indicate that one-third (7/21) of patients with chronic arthralgia followed for over 720 days after CHIKV infection maintained detectable anti-CHIKV IgM in their serum for more than 2 years. Furthermore, of the 8 patients who had a serum sample obtained between 121 and 720 DPSO, 5 (62.5%) remained IgM-positive. One participant, followed for 1,287 DPSO (equivalent to 3.5 years or 43 months), still had anti-CHIKV IgM detectable in their serum. Previous studies have reported long-term IgM persistence following CHIKV infection, with detection at 10 [18], 12 [19], 13 [20, 21], 18 [22], and 35 [9] months, and even up to 13 years post-infection—the longest duration reported in the literature [10]. These observations suggest that the kinetics of anti-CHIKV IgM following infection can vary significantly among individuals and, in some cases, it does not follow the expected pattern of declining to undetectable levels within 3–4 months.

When comparing patients with detectable anti-CHIKV IgM > 720 DPSO to those without detectable IgM, no substantial differences were observed in signs, symptoms, or the levels of IgM and IgG antibodies during the acute phase of illness. This suggests that the clinical characteristics of acute illness are not related to IgM persistence. Since only two convalescent-phase samples were RT-qPCR-positive and all samples collected after 30 DPSO were RT-qPCR-negative, we found no evidence to suggest that persistent viremia is linked to long-term IgM maintenance. Previous studies suggest that CHIKV may evade the immune system and persist in"deep sanctuaries"within joints and tissues, sustaining inflammation [23], which could potentially serve as a stimulus for the continued production of IgM antibodies. Additionally, the possibility of re-exposure to CHIKV cannot be ruled out, given that CHIKV established an endemic-epidemic transmission pattern in Salvador [24]

Our results, which provide evidence for the long-term maintenance of anti-CHIKV IgM, have significant implications for chikungunya diagnosis, patient management, and disease surveillance. In regions that have experienced large CHIKV epidemics or where CHIKV is endemic, serology-based diagnostics may be misleading, as infections from months or even years prior could be interpreted as acute or recent infections. Mistaking past infections for current ones can delay accurate diagnosis, potentially leading to inadequate treatment (i.e. withholding fluid replacement for dengue patients or augmenting the use of anti-inflammatory drugs, which are commonly recommended for post-acute chikungunya-associated joint pain but are contra-indicated in dengue patients due to the risk of bleeding complications). Additionally, patients with positive anti-CHIKV IgM from previous infections might be reported as acute chikungunya cases, misleading the assessment of the dynamics of CHIKV spread. Therefore, we recommend that IgM-based serological diagnosis be interpreted cautiously in regions previously hit by large epidemics or with endemic CHIKV transmission. Wherever possible, RT-PCR should be used as the diagnostic gold standard and paired blood samples should be collected to check for IgM seroconversion, allowing the differentiation between ongoing and prior infections.

This study has several limitations. First, the number and timing of follow-up samples varied among participants; only 30 of the 45 patients had a sample collected after 90 days of symptom onset, and 21 of these had a sample collected after two years. This variability limited the consistent assessment of IgM positivity at each time point. Second, antibody detection depends on the accuracy of the chosen method. In this study, we used ELISA, considered a standard technique for antibody detection. The ELISA test used was designed for the detection of IgM antibodies targeting the CHIKV envelope (E2/E1) proteins and has been validated by the Centers for Disease Prevention and Control (CDC) [25]. We have also evaluated its accuracy, finding it has a sensitivity of 92.4% for convalescent-phase samples from RT-PCR-confirmed CHIKV cases, and a specificity of 97.7% and 90.5% in acute- and convalescent-phase samples from non-CHIKV febrile cases (including dengue and Zika), respectively [26]. Third, the small, non-random convenience sample warrants caution when interpreting P values for the group comparison between patients with and without IgM persistence beyond 720 DPSO. The limited statistical power and potential violations of key assumptions underlying inferential tests reduce the ability to determine if variations are real or incidental. Finally, all patients who had IgM persistence in our study also had chronic arthralgia, so these findings may not apply to patients who fully recover after acute or post-acute disease phases. Future studies should assess whether our results extend to patients without chronic symptoms or to individuals with asymptomatic CHIKV infections. They should also examine if long-term anti-CHIKV IgM correlates with the duration of arthralgia, which could suggest a shared underlying mechanism.

## Conclusion

In conclusion, our findings suggest that patients with chronic arthralgia due to chikungunya exhibit varying rates of decline in anti-CHIKV IgM levels, with some remaining IgM-positive for years after infection. Further studies are needed to clarify the mechanisms underlying the sustained production of anti-CHIKV IgM antibodies at detectable levels. Specifically, studies should examine whether antigenic stimulus, such as viral persistence or CHIKV re-exposure, contributes to this process. Meanwhile, researchers, diagnostic developers, and manufacturers should consider evaluating alternative target proteins or adjusting test cutoffs in IgM-based serological assays to help minimize the detection of IgM signals from past infections. Until IgM tests can accurately differentiate acute from past infections, physicians, laboratory staff and public health professionals from high-burden CHIKV regions should interpret IgM-based serological results for CHIKV with caution.

## Data Availability

The datasets analysed during the current study are available from the corresponding author on reasonable request.
