# Correction: Long-term persistence of serum IgM antibodies against chikungunya virus in patients with chronic arthralgia

**DOI:** 10.1186/s12985-025-02812-9

**Published:** 2025-06-03

**Authors:** Leile Camila Jacob-Nascimento, Rosângela O. Anjos, Moyra M. Portilho, Viviane M. Cavalcanti, Adriane S. Paz, Lorena G. Santos, Moisés S. Sousa, Julia G. Costa, Mariane R. Silva, Patrícia S. S. Moreira, Uriel Kitron, Scott C. Weaver, Mittermayer B. Santiago, Mitermayer G. Reis, Guilherme S. Ribeiro

**Affiliations:** 1 Fundação Oswaldo Cruz, Salvador, Brazil; 2 Escola Bahiana de Medicina E Saúde Pública, Salvador, Brazil; 3 Emory University, Atlanta, USA; 4 World Reference Center for Emerging Viruses and Arboviruses, University of Texas Medical Branch, Galveston, USA; 5 Faculdade de Medicina, Universidade Federal da Bahia, Salvador, Brazil; 6 Yale University, New Haven, USA

**Correction: Virol J 22, 115 (2025)**

**https://doi.org/10.1186/s12985-025–02721-x**

In Table 1 of this article [1], the values in the footnotes a, c, and d were incorrect and their values were updated.

**Table 1:** Demographic, clinical and laboratory features of laboratory-confirmed chikungunya patients

Characteristics	Days post symptoms onset (DPSO) at the time of the visit									
	0–7 (*N* = 45)	10–30 (*N* = 40)	31–60 (*N* = 13)	61–90 (*N* = 7)	91–120 (*N* = 2)	121–720 (*N* = 8)	721–900 (*N* = 17)	901–1080 (*N* = 8)	1,081–1,260 (*N* = 3)	> 1,260 (*N* = 2)
**Demographics and clinical**	**% (n) or median (interquartile range)**									
Sex (female)	33 (73)	30 (75)	11 (84)	4 (57)	1 (50)	7 (87)	12 (70)	6 (75)	2 (66)	1 (50)
Age	44 (35–56)	45 (35–56)	37 (30–49)	56 (47–52)	38 (33–44)	54 (40–68)	44 (33–52)	40 (33–48)	37 (21–55)	44 (33–56)
DPSO	1 (1.5–3)	15 (13–20)	36 (37–44)	74 (67–78)	102 (96 −107)	166 (147–271)	848 (806–860)	1008 (959–1,022)	1,120 (1,120–1172,5)	1,301.5 (1,294–1308,8)
Arthralgia	45 (100)	35 (94) ^*a*^	12 (92)	7 (100)	2 (100)	7 (87)	17 (100)	8 (100)	3 (100)	2 (100)
Myalgia	45 (100)	0 (0) ^*b*^	8 (80) ^*d*^	6 (85)	0 (0)	4 (50)	13 (76)	5 (62)	2 (66)	1 (50)
Joint edema	31 (68)	27 (75) ^*c*^	12 (100) ^*e*^	7 (100)	2 (100)	5 (62)	12 (75)	7 (87)	2 (66)	2 (100)
**Laboratory**										
RT-qPCR CHIKV positive	43 (95)	5 (2)	0 (0)	0 (0)	0 (0)	0 (0)	0 (0)	0 (0)	0 (0)	0 (0)
Cycle Threshold	22.82 (20.0–28.4)	36.75 (35.6–37.9)	-	-	-	-	-	-	-	-
ELISA IgM positive ^*f,h*^	6 (13)	40 (100)	13 (100)	7 (100)	2 (100)	5 (62)	6 (35)	1 (12)	1 (33)	1 (50)
ELISA IgG positive ^*g,h*^	1 (2)	40 (100)	13 (100)	7 (100)	2 (100)	8 (100)	17 (100)	8 (100)	3 (100)	2 (100)

^a^Data available for 37 patients

^b^Data available for 2 patients

^c^Data available for 36 patients

^d^Data available for 10 patients

^e^Data available for 12 patients

^f^Results based on the sample-to-calibrator optical density ratio for the IgM ELISA are interpreted as follows: Negative < 0.9; Positive > 1.1; Indeterminate ≥ 0.9 and ≤ 1.1

^g^Results based on sample-to-calibrator optical density ratio for the IgG ELISA are interpreted as follows: Negative < 0.8; Positive ≥ 1.1; Indeterminate ≥ 0.8 and < 1.1

^h^The Sample/Calibrator ratio is calculated by dividing the optical density obtained with the test sample by the calibrator value, which is unique for the IgG ELISA and an average of a duplicate calibrator for the IgM ELISA

Incorrect footnotes:

^a^Data available for 35 patients

^c^Data available for 27 patients

^d^Data available for 8 patients

Correct Footnote should read:

^a^Data available for 37 patients

^c^Data available for 36 patients

^d^Data available for 10 patients

The original article has been corrected.
